# Sustained Ventricular Tachycardia Presenting As Gastrointestinal (GI) Symptoms

**DOI:** 10.7759/cureus.52494

**Published:** 2024-01-18

**Authors:** Ijeoma C Orabueze, Olushola Ogunleye, Sanjaya Jha

**Affiliations:** 1 Internal Medicine, Vassar Brothers Medical Center Nuvance Health, Poughkeepsie, USA

**Keywords:** myocardial infarction, diarrhea, implantable cardioverter defribillator, vomiting, nausea, sustained ventricular tachycardia

## Abstract

Ventricular arrhythmias (VAs) are among the most common cardiac rhythms seen among patients. Patients presenting with frequent, sustained ventricular tachycardia (VT) pose a dilemma for clinicians due to the constant risk of sudden cardiovascular compromise.

Ventricular tachycardia, which is commonly seen in patients with defects in cardiac anatomy, has been associated with an increased risk of sudden death. A previous myocardial scar from a previous myocardial infarction remains the most common cause of sustained monomorphic VT (SMVT) in patients with structural cardiac disease.

Studies have shown that implantable cardioverter-defibrillators (ICDs) can be used for primary prevention in patients with ischemic or nonischemic cardiomyopathy whose ejection fraction remains below 35% despite guideline-directed medical therapy. It can also be used for secondary prevention of sudden cardiac death in patients who have had a VT or ventricular fibrillation (VF). Identifying individuals at risk for developing deleterious VTs who will benefit from ICD placement for prevention has been the objective of many large studies in recent years. We present a case of clinical importance involving the use of ICD in the primary prevention of mortality from sustained ventricular arrhythmias.

## Introduction

Sustained ventricular tachycardia (VT) is defined as a regular, wide complex tachycardia that lasts greater than 30 seconds [1]. Symptoms include chest discomfort, palpitations, dizziness, and syncope [2]. In rare presentations, gastrointestinal symptoms such as nausea, vomiting, and diarrhea are harbingers of sustained VT [2]. We present the case of a 53-year-old male who presented with the above gastrointestinal (GI) symptoms and was found to be in sustained VT.

## Case presentation

**Figure 1:**
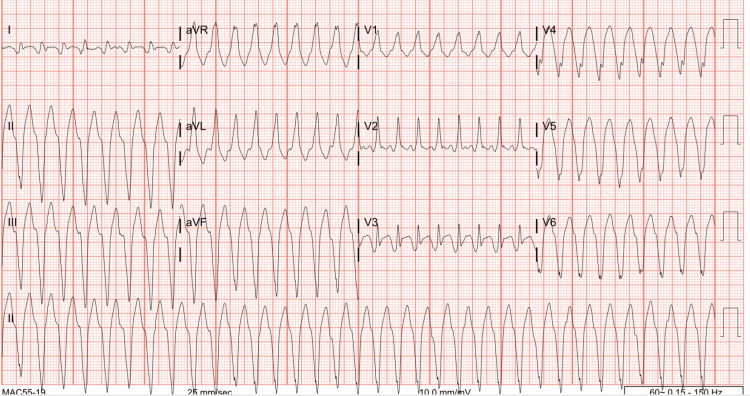
The ECG on presentation Monomorphic ventricular tachycardia with rates in the 200s

We present the case of a 53-year-old male with a medical history of hypertension, hyperlipidemia, ischemic cardiomyopathy with ejection fraction <20%, and coronary artery disease status post-myocardial infarction requiring percutaneous coronary intervention with a left anterior descending stent placed 10 years earlier, then lost to follow-up due to insurance issues. His home medications were aspirin and atorvastatin. He did not have an implantable cardioverter defibrillator at the time of presentation, as he had not seen a cardiologist in over 10 years due to the lack of insurance. He presented with a two-day history of shortness of breath, nausea, vomiting, and diarrhea. On presentation, he was hypotensive (82/69 mmHg), tachycardic (211 beats/min), and tachypneic (30 breaths/min), with electrocardiogram findings of monomorphic ventricular tachycardia and a heart rate in the 200s (Figure 1).

His electrolytes on presentation were within normal limits (Table 1).

**Table 1:** Laboratory values on presentation Numbers in brackets are the normal reference ranges.

Lab test	Results (Reference range)
Potassium	4.0 mmol/l (3.5-5.0 mmol/l)
Magnesium	2.0mg/dl (1.6-2.6 mg/dl)
Calcium	9.4 mg/dl (8.6-10.4 mg/dl)
Sodium	139 mmol/l (135-145 mmol/l)

**Figure 2:**
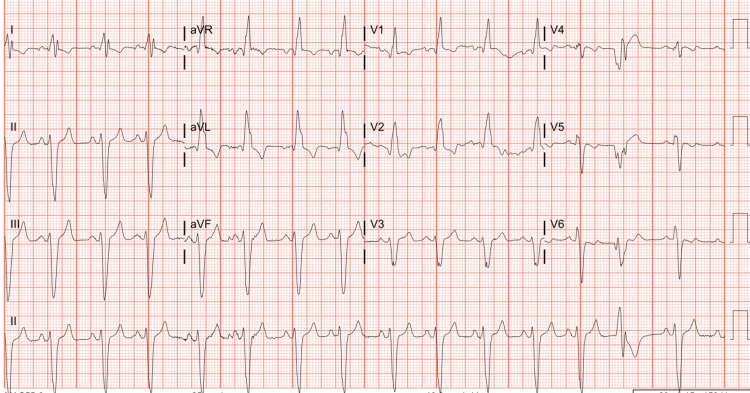
The ECG after cardioversion Sinus rhythm with the right bundle branch block

He underwent emergent unsynchronized electrical cardioversion with conversion back to sinus rhythm (Figure 2) and maintained sinus rhythm with intravenous amiodarone.

**Figure 3:**
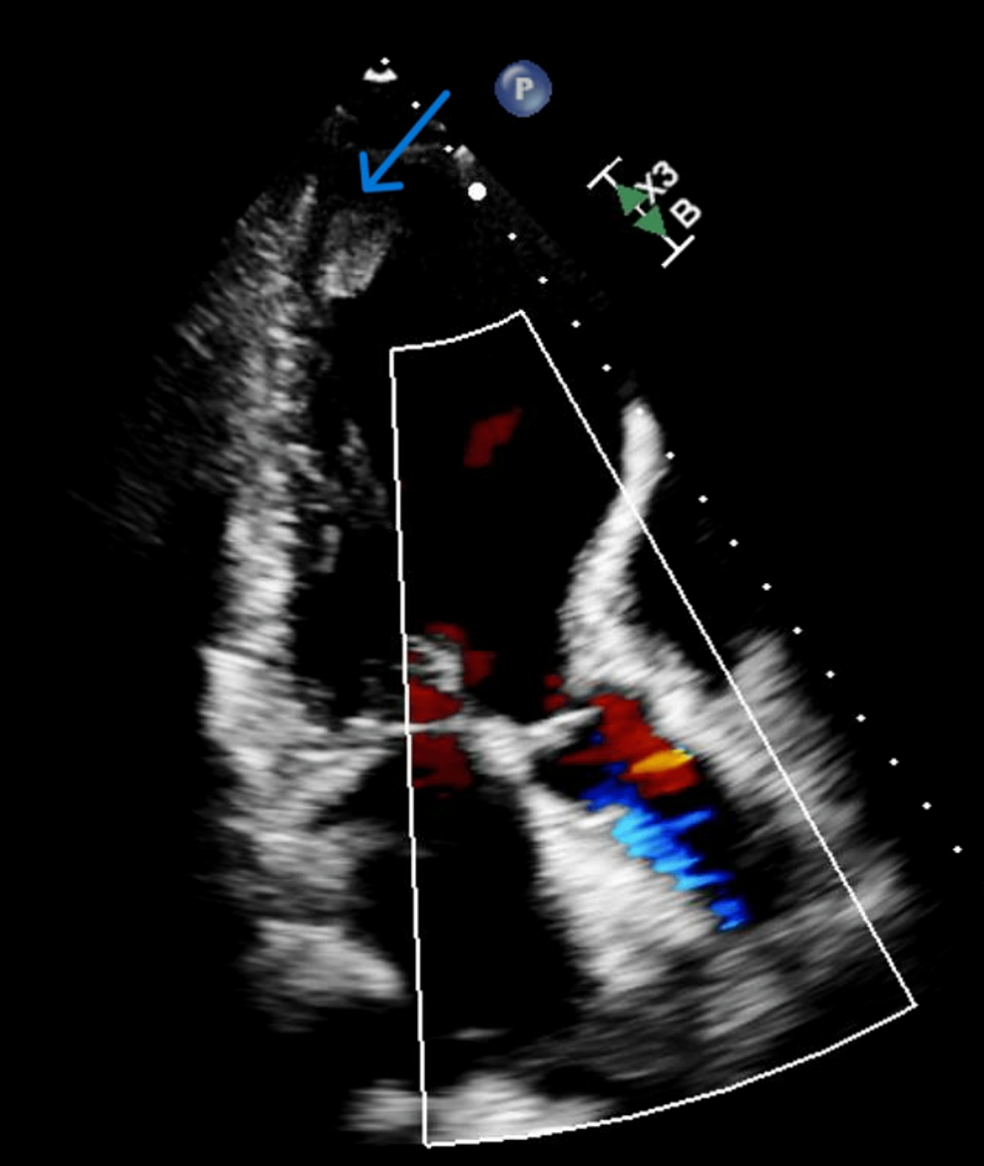
Transthoracic echocardiogram The blue arrow points to a large thrombus at the apex of the left ventricle.

A transthoracic echocardiogram revealed a large thrombus in the apex of the left ventricle (Figure 3).

**Figure 4:**
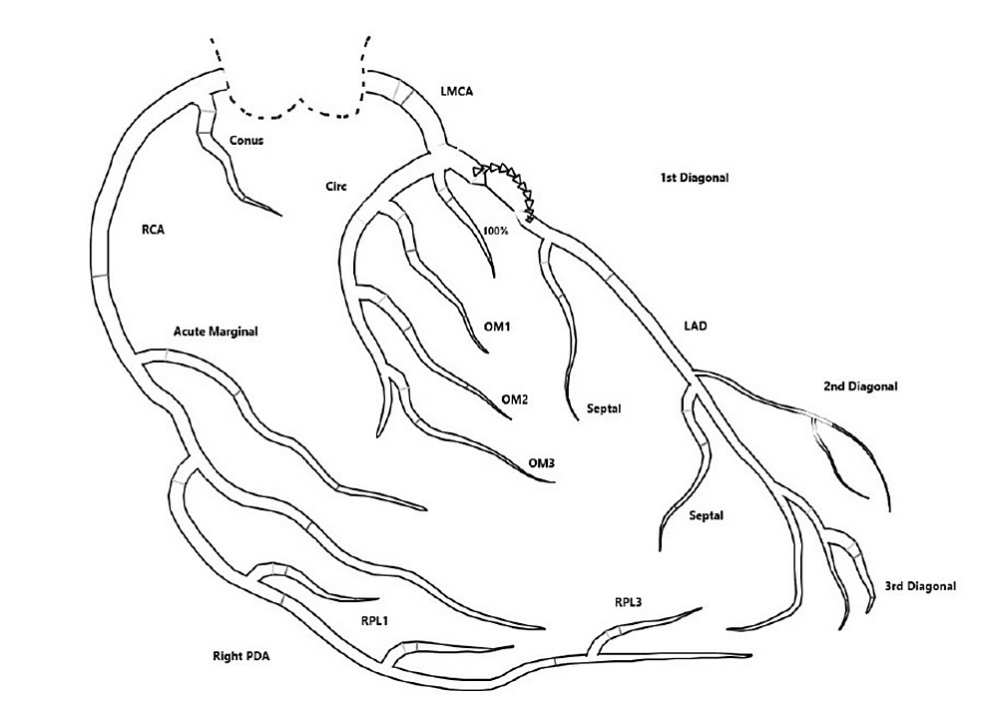
Left cardiac catheterization There is a discrete 100% proximal lesion in the left anterior descending artery (LAD) and collateral flow from LAD to LAD. Otherwise, there is no angiographically significant disease. RCA: right coronary artery; LMCA: left main coronary artery; OM: obtuse marginal; LAD: left anterior descending artery; PDA: patent ductus arteriosus; RPL: right posterolateral Figure credit: Orabueze Ijeoma

Left cardiac catheterization (Figure 4) revealed a discrete 100% proximal lesion in the left anterior descending (LAD) artery with collateral flow from the proximal LAD to the distal LAD, so no stents were placed.

The infectious diseases department was consulted to rule out the infectious etiology of his GI symptoms, but his symptoms had resolved within 12 hours of his arrival at the hospital. He was afebrile and had mild leukocytosis on his labs, which were deemed due to hemoconcentration from dehydration vs. reactivity in the setting of cardiac stress. Prior to discharge, he underwent an implantable cardioverter defibrillator (ICD) placement and was commenced on warfarin, carvedilol, entresto, and furosemide.

## Discussion

Coronary artery disease, with a history of myocardial infarction, is the most common underlying heart disease associated with the development of sustained monomorphic ventricular tachycardia (SMVT) and ventricular fibrillation (VF) [3]. Like in our patient, the most common underlying mechanism is the generation of a reentry circuit within the scar tissue formed at the site of prior myocardial infarction [4]. Sustained VT is defined as a regular, wide-complex tachycardia originating from the ventricles and lasting 30 seconds or resulting in hemodynamic instability requiring immediate unsynchronized cardioversion [1]. Ventricular tachycardia frequently occurs among patients with structural heart disease (cardiomyopathies) or channelopathies (such as long QT syndrome and Brugada syndrome) [5]. Risk factors for VT include a prior history of hypertension, prior myocardial infarction, ischemic ST-segment changes at presentation, and chronic obstructive pulmonary disease. There are two forms of VT: monomorphic and polymorphic. Sustained VT that is not associated with acute coronary syndrome is often monomorphic, as it is usually due to scar-related reentry but may degenerate into ventricular fibrillation [6]. Monomorphic VT, occurring in the absence of structural heart disease, is commonly referred to as idiopathic VT. Polymorphic VT can occur in the absence of structural heart disease and may be idiopathic or due to a myocardial channelopathy or drug-induced long QT syndrome [6].

Sustained monomorphic VT is the most common arrhythmic cause of out-of-hospital cardiac arrest [6]. Due to the lethality of the condition, a brief, immediate assessment of symptoms, vital signs, and level of consciousness is essential. Hence, this case report aims to educate on the rare GI symptoms that monomorphic VT can present with and facilitate quick patient assessment and cardioversion as indicated. It is, therefore, imperative to study the different symptoms that have been associated with this arrhythmia, as little is known about the various presenting symptoms [6]. During episodes of VT, common symptoms include palpitations, chest tightness, and syncope [4]. A study performed by Habakuk et al. [2] analyzed the clinical symptoms of VT in patients who did not experience a cardiac arrest and found chest pain (25% in males, 0% in females), chest discomfort (11% in males, 33.3% in females), dyspnea (11% in males, 1% in females), palpitations (4% in males, 1% in females), dizziness (15% in males, 0% in females), and syncope (9% in males, 0% in females) [2]. Other than the aforementioned symptoms, GI symptoms like nausea, vomiting, and diarrhea could indicate a VT, like in our patient, adding to the quality of the history taking. Our patient's symptoms had resolved within 12 hours of arrival. He was evaluated by the infectious diseases department and any underlying infectious cause was ruled out. His symptoms never returned while he was hospitalized.

To our knowledge, there are no previous reports in adults of an association between VT and GI symptoms except when there was electrolyte derangement. Our patient had no electrolyte abnormalities (Table 1) attributable to his GI symptoms, like hypokalemia or hypomagnesemia, that could have precipitated his VT. Electrocardiography remains the gold standard for diagnosing VT, showing a rapid, regular tachycardia with QRS complexes of greater than normal width (>120 ms) and having the appearance of either a right or left bundle branch block. The complexes may have constant (monomorphic) or changing (polymorphic) forms [7]. Patients who are hemodynamically unstable or have pulseless VT require emergent defibrillation and cardiopulmonary resuscitation (CPR) as per Advanced Cardiovascular Life Support (ACLS) guidelines. Hemodynamically unstable patients with a pulse require urgent cardioversion, and hemodynamically stable patients with SMVT require intravenous antiarrhythmics for pharmacological cardioversion [8]. Our patient was hemodynamically unstable, necessitating immediate cardioversion at presentation. When all reversible and identifiable causes of SMVT have been ruled out, nearly all patients require ICD placement, as it reduces the risk of sudden cardiac death, treats any recurring VTs, and reduces the overall mortality risk. Some patients with heart failure symptoms who require an ICD may qualify for a cardiac resynchronization therapy-defibrillator (CRT-D) if they have concurrent QRS of 150ms or more and a left bundle branch block. However, the indication for an ICD in our patient was for primary prevention, as he had a myocardial infarction 10 years prior. He was not optimized on guideline-directed medical therapy, as he never followed up due to insurance issues. An ICD reduces mortality in patients at risk for sustained ventricular arrhythmia, primarily by delivering high-voltage shocks that terminate potentially fatal ventricular arrhythmias or by applying a burst of rapid pacing that interrupts reentry [9, 10].  

## Conclusions

Sustained VT remains an important cause of sudden cardiac deaths, but it also has less fatal presentations such as palpitations and syncopal episodes. Worthy of note are rarer presentations such as nausea, vomiting, and diarrhea. Depending on the patient’s clinical presentation and hemodynamic stability, therapy ranges from pharmacologic to electrical cardioversion, while intervention by placing an ICD has been shown to reduce mortality in these groups of patients.

## References

[1] Piccini JP, Schulte PJ, Pieper KS (2011). Antiarrhythmic drug therapy for sustained ventricular arrhythmias complicating acute myocardial infarction. Crit Care Med.

[2] Havakuk O, Viskin D, Viskin S (2020). Clinical presentation of sustained monomorphic ventricular tachycardia without cardiac arrest. J Am Heart Assoc.

[3] Hadid C (2015). Sustained ventricular tachycardia in structural heart disease. Cardiol J.

[4] Benito B, Josephson ME (2012). Ventricular tachycardia in coronary artery disease [Article in Spanish]. Rev Esp Cardiol.

[5] Samuel M, Elsokkari I, Sapp JL (2022). Ventricular tachycardia burden and mortality: association or causality?. Can J Cardiol.

[6] Koplan BA, Stevenson WG (2009). Ventricular tachycardia and sudden cardiac death. Mayo Clin Proc.

[7] Kanagasundram AN, Richardson TD, Stevenson WG (2021). The heart rate of ventricular tachycardia. Circulation.

[8] Buxton A (2023). Sustained Monomorphic Ventricular Tachycardia in Patients With Structural Heart Disease: Treatment and Prognosis. UpToDate. UpToDate.

[9] Stevenson WG (2009). Ventricular scars and ventricular tachycardia. Trans Am Clin Climatol Assoc.

[10] Connolly SJ, Dorian P, Roberts RS (2006). Comparison of beta-blockers, amiodarone plus beta-blockers, or sotalol for prevention of shocks from implantable cardioverter defibrillators: the OPTIC study: a randomized trial. JAMA.

